# Allosteric inhibition of the guanine nucleotide exchange factor DOCK5 by a small molecule

**DOI:** 10.1038/s41598-017-13619-2

**Published:** 2017-10-31

**Authors:** Yann Ferrandez, Wenhua Zhang, François Peurois, Lurlène Akendengué, Anne Blangy, Mahel Zeghouf, Jacqueline Cherfils

**Affiliations:** 1 Laboratoire de Biologie et Pharmacologie Appliquée, CNRS and Ecole Normale Supérieure Paris-Saclay, Cachan, France; 2 Centre de Recherche de Biochimie Macromoléculaire, CNRS and Université Montpellier, Montpellier, France

## Abstract

Rac small GTPases and their GEFs of the DOCK family are pivotal checkpoints in development, autoimmunity and bone homeostasis, and their abnormal regulation is associated to diverse pathologies. Small molecules that inhibit their activities are therefore needed to investigate their functions. Here, we characterized the mechanism of inhibition of human DOCK5 by C21, a small molecule that inhibits mouse Dock5 in cells and blocks bone degradation in mice models of osteoporosis. We showed that the catalytic DHR2 domain of DOCK5 has a high basal GEF activity in the absence of membranes which is not regulated by a simple feedback loop. C21 blocks this activity in a non-competitive manner and is specific for DOCK5. In contrast, another Dock inhibitor, CPYPP, inhibits both DOCK5 and an unrelated GEF, Trio. To gain insight into structural features of the inhibitory mechanism of C21, we used SAXS analysis of DOCK5^DHR2^ and crystallographic analysis of unbound Rac1-GDP. Together, these data suggest that C21 takes advantage of intramolecular dynamics of DOCK5 and Rac1 to remodel the complex into an unproductive conformation. Based on this allosteric mechanism, we propose that diversion of intramolecular dynamics is a potent mechanism for the inhibition of multidomain regulators of small GTPases.

## Introduction

DOCK (dedicator of cytokinesis) proteins activate Rho family GTPases to regulate development, autoimmunity and bone homeostasis and they are involved in cancer and severe developmental and immune-related diseases (reviewed in refs^1–3^). DOCK proteins are characterized by the presence of a DHR2 domain of about 400 amino acids that carries the GDP/GTP nucleotide exchange (GEF) activity, and an upstream DHR1 domain that binds membrane phosphoinositides. Additional domains in N- and C-terminus define several subfamilies, including the DOCK A subfamily (Dock1/180, Dock2 and Dock5) which is characterized by an SH3 domain in N-terminus (reviewed in ref.^2^).

**Figure 1:**
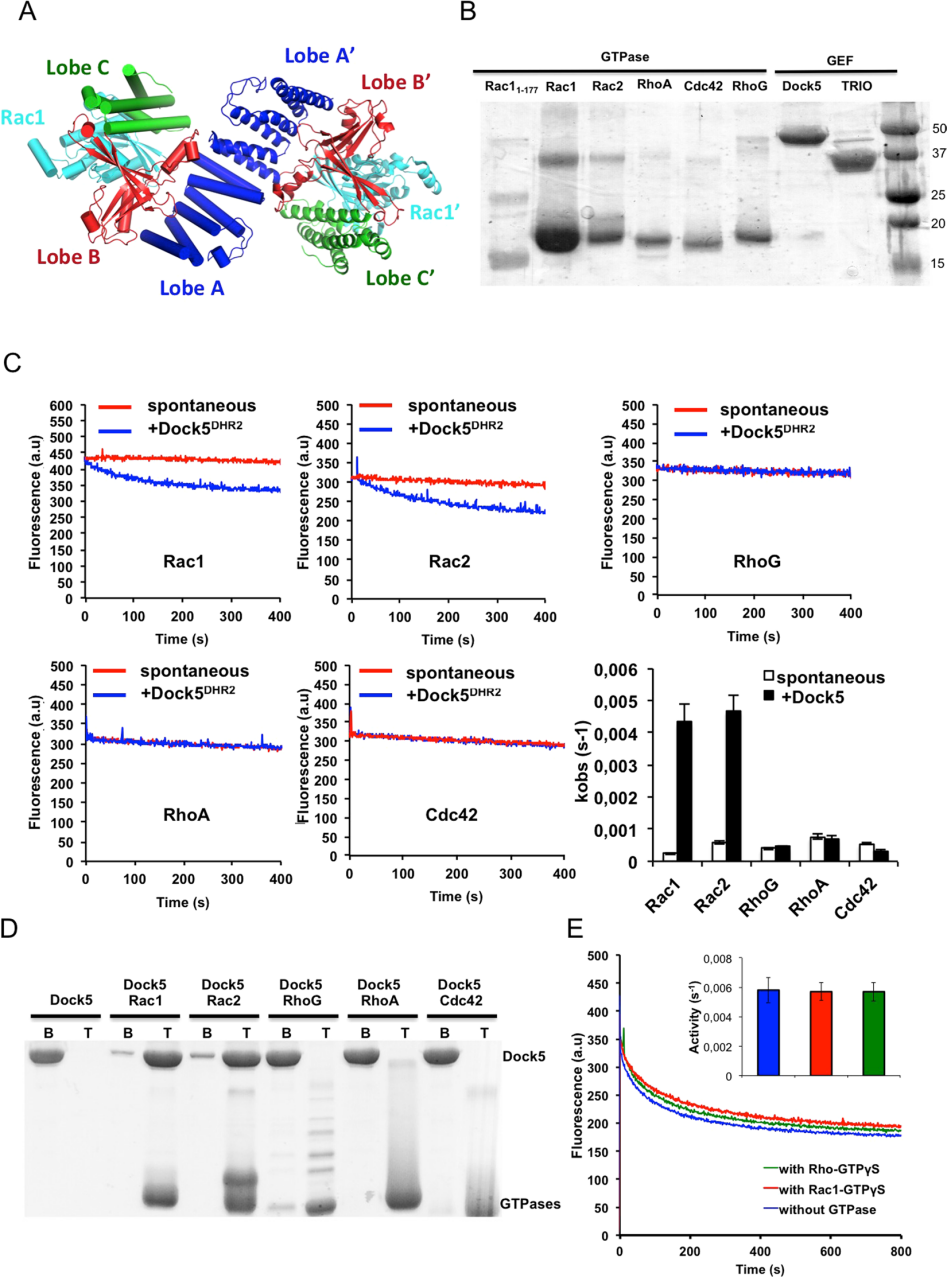
Activity and regulation of human DOCK5^DHR2^. (**A**) Architecture of Dock^DHR2^-GTPase complexes. The structure is from the Dock2^DHR2^-Rac1 complex (PDB entry 2YIN, ref.^4^). Individual monomers in Dock2^DHR2^ are identified by cartoon and cylindrical representations with lobes A, B and C colored in blue, red and green respectively. The Rac GTPase is in cyan. (**B**) SDS-PAGE analysis of protein constructs used in this study. (**C**) Fluorescence kinetic traces of the activation of Rac family GTPases by DOCK5^DHR2^. K_obs_ values (s^−1^) are shown in the histogram. (**D**) Recruitment of DOCK5^DHR2^ to liposomes in the presence and absence of GTPases, analyzed by liposome flotation. GTPases were loaded with GDP and tethered to liposomes by a C-terminal His-tag. B, bottom fraction containing soluble proteins. T, top fraction containing liposome-bound proteins. (**E**) Fluorescence kinetic traces of the activation of Rac1 by DOCK5^DHR2^ measured after pre-incubation with GTPγS-bound GTPases as indicated.

Crystal structures of DHR2/GTPase complexes have been solved for Dock9/Cdc42^4^ and Dock2/Rac1^5,6^ and for a truncated DHR2 domain from Dock8 in complex with Cdc42^7^. These structures identified key features of DHR2 domains and provided a description of the steps involved in GDP dissociation and GTP reloading. They showed that DHR2 is a symmetrical dimer comprised of lobe A, which mediates dimerization, and lobes B and C, which generate the catalytic center and interact with the nucleotide-sensing switch regions of the GTPase (Fig. 1A). Lobe B forms extensive interactions with the switch 1 that pries it open, while lobe C binds to the switch 2 and carries a nucleotide sensor loop that inserts into the nucleotide-binding site to dissociate GDP. Our understanding of the regulation of the DHR2 domain at the biochemical and structural levels has in contrast remained more fragmentary. Intramolecular autoinhibitory interactions between N-terminal regions and the DHR2 domain have been reported for several DOCK family members^8–10^. In addition, it has been reported that Dock11 is regulated by a positive feedback loop^11^ and that efficient GEF activity of the DHR2 domain of Dock7 requires the lipid-modified C-terminus of Cdc42 to be attached to membranes^12^.

Targeting GEFs by chemical compounds is an important path to discovering drugs that block small GTPases signaling in diseases (reviewed in ref.^13^). There is mounting evidence that DOCK family members are pivotal nodes in Rac family signaling in bone and immune diseases, which calls for the discovery of chemical inhibitors that could pave the way for therapeutic intervention. Recent studies identified two chemical leads that block the activation of Rac1 by members of the DOCK A subfamily. C21 was discovered using a yeast-based 3-hybrid screen^14^, in which activation of Rac1 by mouse Dock5 and binding of Rac1-GTP to an effector are simultaneously reconstituted in yeast^15^. C21 inhibited the Dock5-dependent formation of the sealing zone, where the bone matrix is resorbed in osteoclasts, and it protected mice against bone degradation in models of bone metastasis, menopause and rheumatoid arthritis^16^. An unrelated chemical compound, CPYPP, was identified in a screen for disruptors of the interaction between recombinant Dock2 and Rac1, and effectively inhibited Rac activation in lymphocytes resulting in the reduction of chemotactic response and T cell activation^17^. However, the mechanisms whereby these compounds block DOCK proteins are not known.

Here we focused on the DHR2 domain of human DOCK5 and analyzed the mechanisms of its inhibition by these chemical compounds. First, we established reference activities for DOCK5^DHR2^, showing that it activates Rac but no other related GTPases, and that this high basal activity is independent of the tethering of Rac1 to membranes and is not regulated by a feedback loop. We then showed that C21 inhibits this activity in a non-competitive manner and this inhibition is specific for DOCK5. In contrast, CPYPP inhibits both DOCK5 and Trio, an unrelated GEF that belongs to the DH domain-containing RacGEF family. Finally, we used SAXS analysis of DOCK5^DHR2^ and crystallographic analysis of unbound Rac1-GDP to gain insight into structural features of the inhibitory mechanism of C21. Small-Angle X-ray Scattering (SAXS) is a powerful method to analyze structural determinants such as flexibility or disorder in proteins, which are pivotal for function and inhibition but are difficult to observe by other methods (reviewed in refs^18–20^). We found that unbound DOCK5^DHR2^ resembles the dimeric structure of Dock2^DHR2^ and that it displays conformational dynamics that diminishes upon binding of Rac1. Together, our data suggest a model in which C21 takes advantage of the intramolecular dynamics of DOCK5 and Rac1-GDP to remodel the complex in a conformation that cannot proceed to GDP dissociation. Based on this mechanism, we propose that diversion of intramolecular dynamics can be exploited by chemical compounds to remodel protein complexes into unproductive conformations, and has the potential for a variety of applications to the inhibition of GEFs and multidomain proteins in diseases.

## Results

### Specificity and regulation of the DHR2 domain of human DOCK5

In order to establish reference values for the analysis of inhibitors, we assessed the GEF efficiency and regulation of the DHR2 domain of human DOCK5 (residues 1212–1642, DOCK5^DHR2^ hereafter) towards human Rac1, Rac2, RhoA, Cdc42 and RhoG, using highly purified recombinant proteins (Fig. 1B). The kinetics of nucleotide exchange was monitored in solution in the presence of catalytic amounts of DOCK5^DHR2^, by following the decay in fluorescence associated to the replacement of Mant-GDP by GTP. DOCK5^DHR2^ efficiently activated Rac1 and Rac2 but was inactive towards Cdc42, RhoA and RhoG (Fig. 1C). k_cat_/K_M_ values determined over a range of catalytic DOCK5^DHR2^ concentrations were 3 × 10^4^ M^−1^s^−1^ for Rac1 and 3.2 × 10^4^ M^−1^s^−1^ for Rac2, which is similar to the activity of mouse DOCK5^DHR2^ ^16^ and is in the range found for GEF domains for other small GTPases (for example, refs^21,22^). These activities, which are higher than those reported for DHR2 domains from other DOCK proteins^5,11,12^, may represent the basal rate of DHR2 domains in solution. Recently, GEFs from various families have been reported to be directly activated by their own GTP-bound substrates (reviewed in ref.^23^); notably, a positive feedback effect acting directly on the DHR2 domain has been described for Dock7^12^. We investigated the interaction of DOCK5^DHR2^ with nucleotide-bound Rac1 by different assays. First, we observed that DOCK5^DHR2^ interacts with GDP-bound and GTP-bound Rac1 using size exclusion chromatography (Figure S1). We confirmed this interaction in a liposome flotation assay, which showed that DOCK5^DHR2^ alone is soluble but is entirely recruited to liposomes in the presence of liposome-tethered, full-length Rac1 and Rac2 (Fig. 1D). This effect was not observed for liposome-tethered, full-length RhoG, RhoA or Cdc42. Finally, we investigated feedback effects by incubating DOCK5^DHR2^ with excess Rac1-GTPγS (a non hydrolyzable GTP analog) prior to the exchange reaction. The exchange rate was not modified by Rac1-GTPγS, indicating that DOCK5^DHR2^ is not regulated by a direct feedback effect (Fig. 1E). We also did not observe a difference in GEF activity whether DOCK5^DHR2^ was assayed in solution or with liposome-tethered Rac1 (not shown).

We conclude that human DOCK5^DHR2^ activates Rac subfamily GTPases with a high specificity and that, in contrast to most known GEFs which interact strongly only with nucleotide-free GTPases (reviewed in ref.^24^), it interacts strongly with both nucleotide-free and nucleotide-bound Rac. However this strong interaction with Rac-GTP does not implement a simple feedback effect and probably rather reflects the unique GEF mechanism of the DHR2 domain resolved by crystal structures^4^. The fact that DOCK5^DHR2^ is inactive towards RhoG also rules out an indirect positive feedback effect in which it would produce RhoG-GTP, which is a known upstream activator of Dock180 acting through Elmo1^25^ and possibly of other Dock A subfamily members. We observed that DOCK5^DHR2^ is not regulated by a direct interaction with membranes and that the C-terminus of Rac1 does not play a direct role in the nucleotide exchange reaction. Together, these experiments provide accurate reference rates for DOCK5^DHR2^ and they also indicate that its structure and inhibition can reliably be investigated in solution in the absence of other components.

### Characterization of the inhibition of DOCK5^DHR2^ by small molecules

**Figure 2:**
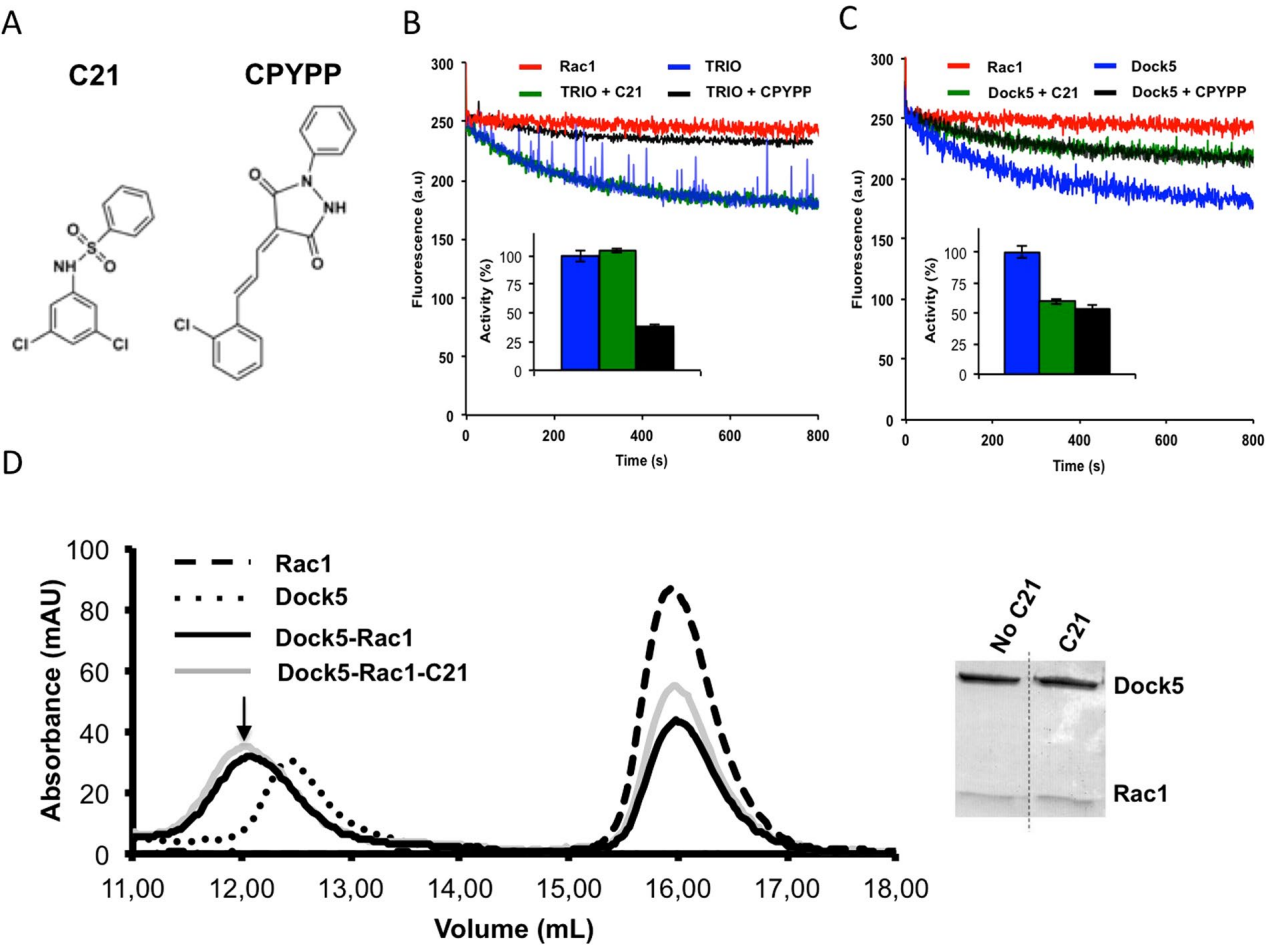
Inhibition of DOCK5 by C21 and CPYPP (**A**) Chemical structures of CPYPP and C21. (**B**) Inhibition of Trio^DH1-PH1^ by CPYPP and C21 analyzed by fluorescence kinetics. (**C**) Inhibition of DOCK5^DHR2^ by CPYPP and C21 analyzed by fluorescence kinetics. (**D**) Effect of C21 on the formation of the Rac1-DOCK5^DHR2^ complex analyzed by size exclusion chromatography. Right panel: The peaks containing the complexes (indicated by an arrow) were analyzed by SDS-PAGE.

Two chemical compounds of unrelated chemical structure, C21 and CPYPP (Fig. 2A), have recently been reported to inhibit DOCK5^16^ and Dock2 and other Dock A subfamily members^17^, respectively. We used fluorescence kinetics to determine the efficiency and specificity of C21 and CPYPP towards DOCK5^DHR2^ (Fig. 2C) and Trio^DH1−PH1^, an unrelated Rac1GEF with a Dbl homology (DH) domain (Fig. 2B). Both C21 and CPYPP efficiently inhibit the activation of Rac1 by DOCK5^DHR2^. C21 does not inhibit the activation of Rac1 by Trio^DH1−PH1^, indicating that it is specific for the DOCK family. Unexpectedly, CPYPP inhibits Trio^DH1−PH1^, indicating that it is not specific for Dock family GEFs and may function by exerting a direct effect on Rac1. We note that this observation is in contrast to previous reports^17^, possibly arising from different experimental setups. Because CPYPP is not GEF-specific, we focused on C21 for the rest of the analysis. To get further insight into the inhibitory mechanism of C21, we analyzed its effect on the formation of the Rac1-DOCK5^DHR2^ complex using size exclusion chromatography. As shown in Fig. 2D, C21 does not disrupt the formation of the complex. Together, these data indicate that C21 discriminates between the DHR2- and DH-containing RacGEF families, and that it functions by a non-competitive mechanism in which the GEF activity of DOCK5 is impaired but not the ability of DOCK5 to bind Rac1.

### Structural dynamics of DOCK5 and Rac1 support the allosteric mechanism of C21

To investigate structural features in DOCK5 and Rac1 that could be susceptible to allosteric modulation by C21, we used synchrotron SAXS coupled to size exclusion chromatography (SEC-SAXS) to analyze the structure of DOCK5^DHR2^ and of the DOCK5^DHR2^-Rac1 complex in solution, and X-ray crystallography to determine the structure of Rac1-GDP.

**Figure 3:**
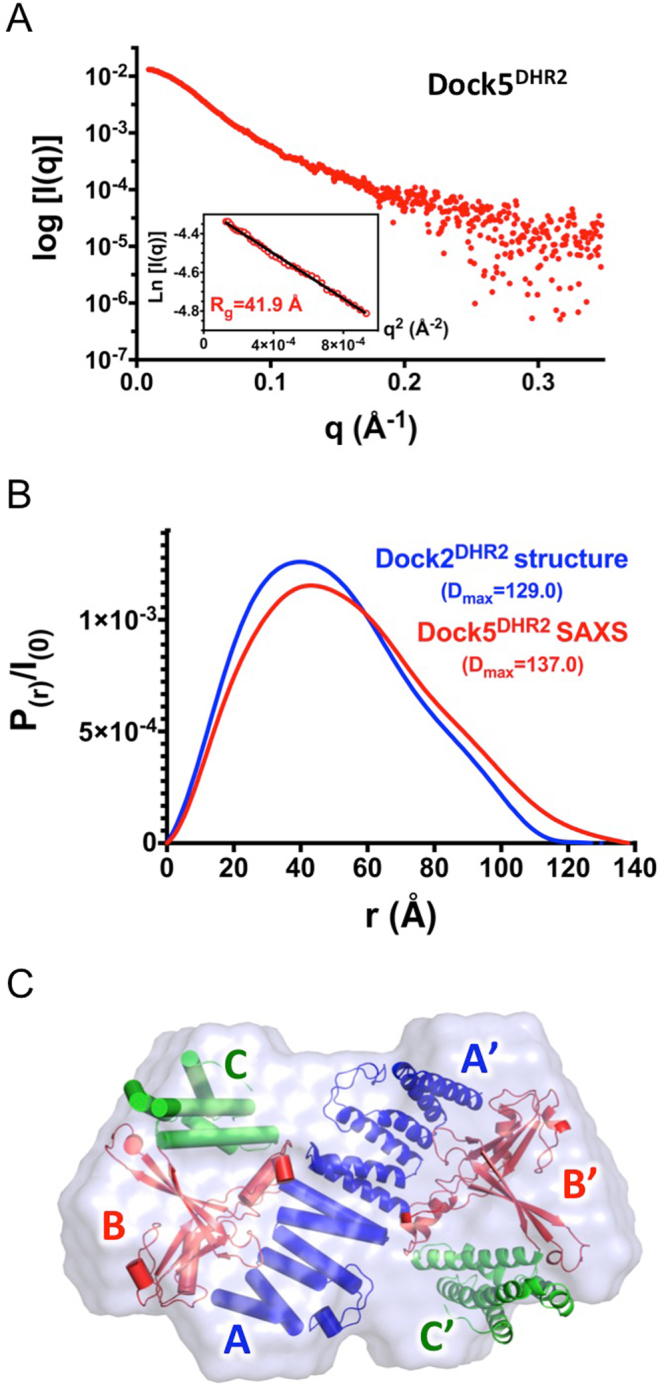
SEC-SAXS analysis of unbound DOCK5^DHR2^. (**A**) SAXS profile of DOCK5^DHR2^. The insert shows the Guinier plot (q_max_*R_g_ = 1.28) and the estimation of R_g_ from Guinier analysis. (**B**) Comparison of the distance distribution function I_(r)_/I_(0)_ plots of DOCK5^DHR2^ experimental SAXS data (in red) and of Dock2^DHR2^ crystal structure (in blue; derived from the Dock2^DHR2^-Rac1 crystal structure, PDB: 3B13). The D_max_ estimated from the DOCK5^DHR2^ SAXS data and calculated from the Dock2^DHR2^ crystal structure are indicated. (**C**) Surface representation of the DOCK5^DHR2^ model calculated with DAMMIF. The χ^2^ value of the DAMMIF model to the experimental amplitudes is 1.5; the χ^2^ value of the Dock2^DHR2^ crystal structure to the DAMMIF model is 2.5. The crystal structure of the Dock2^DHR2^ dimer was superposed onto the DOCK5^DHR2^ envelope using SUPCOMB. Dock2^DHR2^ is represented as in Fig. 1A.

All SAXS data acquisition and analyses are summarized in Table 1. The scattering profile of DOCK5^DHR2^ is shown in Fig. 3A. Interestingly, we noted that although the protein eluted as a single, well-resolved I_(0)_ peak, the radius of gyration (R_g_, an indicator of the overall spread of the protein) slightly decreased across the frames (Figure S2A). The frames before, at the middle of, and after the peak were therefore grouped and analyzed separately. The frames before the peak yielded an R_g_ value of 42 Å, a distance distribution function P_(r)_ with a maximal dimension (D_max_) of 137 Å (Fig. 3B) and a Kratky plot representative of a well-folded protein (Figure S2B). We calculated an atomic model of the DOCK5 monomer with I-Tasser^26^ and made it dimeric using the crystallographic Dock2 dimer as a template (see Fig. 1A). Comparison with CRYSOL of the experimental scattering amplitudes with amplitudes calculated with the DOCK5 model (q = 0.35 Å^−1^) gave a χ^2^ value of 2.6, which could be lowered to 1.2 by rigid body refinement of the C-lobe using Dadimodo^27^. By comparison, fitting of the related crystallographic Dock9 dimer gave a χ^2^ value of 4.6 after refinement with Dadimodo. Imposing P2 symmetry, we constructed 20 SAXS envelopes of unbound DOCK5^DHR2^ with DAMMIF and chose the clustered envelopes with the lowest Normalized Spatial Discrepancy (NSD) to represent the DOCK5^DHR2^ dimer in solution. Because of its symmetry and elongated shape, the DOCK5^DHR2^ dimer was readily fitted into this envelope and the individual A, B and C lobes of both subunits were located without ambiguity (Figs 3C and S2C). These data indicate that the frames before the peak depict a conformation of DOCK5 that closely resembles the structure of the crystallographic Dock2 dimer. Fitting the DOCK5 model to the frames located in the middle of, and after, the peak gave χ^2^ values of 7.9 and 5.1 respectively. These values are in the range obtained when fitting the Dock9 dimer to the frames before the peak, indicating that the conformations of DOCK5 in the frames from the middle of and after the peak have structural differences with the previous frames that are of the same range as those between DOCK5 and Dock9. Together, these observations suggest that DOCK5^DHR2^ exists as a conformational population, in which a conformation similar to that of the Dock2 dimer can readily be identified.

**Table 1:** Statistics and summary of the SEC-SAXS data analysis.

Data Collection Parameters by in-line HPLC-coupled SAXS			
**HPLC(SEC Column)**	Bio-SEC. 3 (Agilent)		
**SAXS Instrument**	SWING beamline (PCCD170170 Aviex Detector)		
**Beam Geometry**	Pinhole		
**Wavelength (Å)**	1.033		
**q Range (Å** ^**−1**^ **)**	0.006–0.5		
**Exposure Time (s)**	2.0 per frame		
**Temperature (K)**	295		
**Structural Parameters**		**DOCK5** ^**DHR2**^ *	**DOCK5** ^**DHR2**^ **-Rac1**
**Guinier Fit**	***I***_**(0)**_ **(cm** ^−**1**^ **)**	0.14 ± 3.6e-6**	0.45 ± 5.2e-6
	**R**_**g**_ **(Å)**	41.8 ± 3.8e-1	45.7 ± 1.6e-1
**P**_**(r)**_	**R**_**g**_ **(Å)**	42.0	46.7
	**D**_**max**_ **(Å)**	137.0	155.0
**Molar Mass Determination (kD)**			
**Calc**. **from sequence**		102.2	141.5
**Esti**. **by ScÅtter**		110.0	140.0
**Molecular Shape Modeling**			
**DAMMIF**	χ^2^	1.5	1.6
**NSD**		1.08 ± 1.6e-1	1.08 ± 2.6e-1

*This analysis was carried out with frames located before the peak. **The lower I_(0)_ is due to a difference in the volume injected onto the HPLC-SEC column, resulting in a 3.5 lower concentration of the sample at the elution peak.

**Figure 4:**
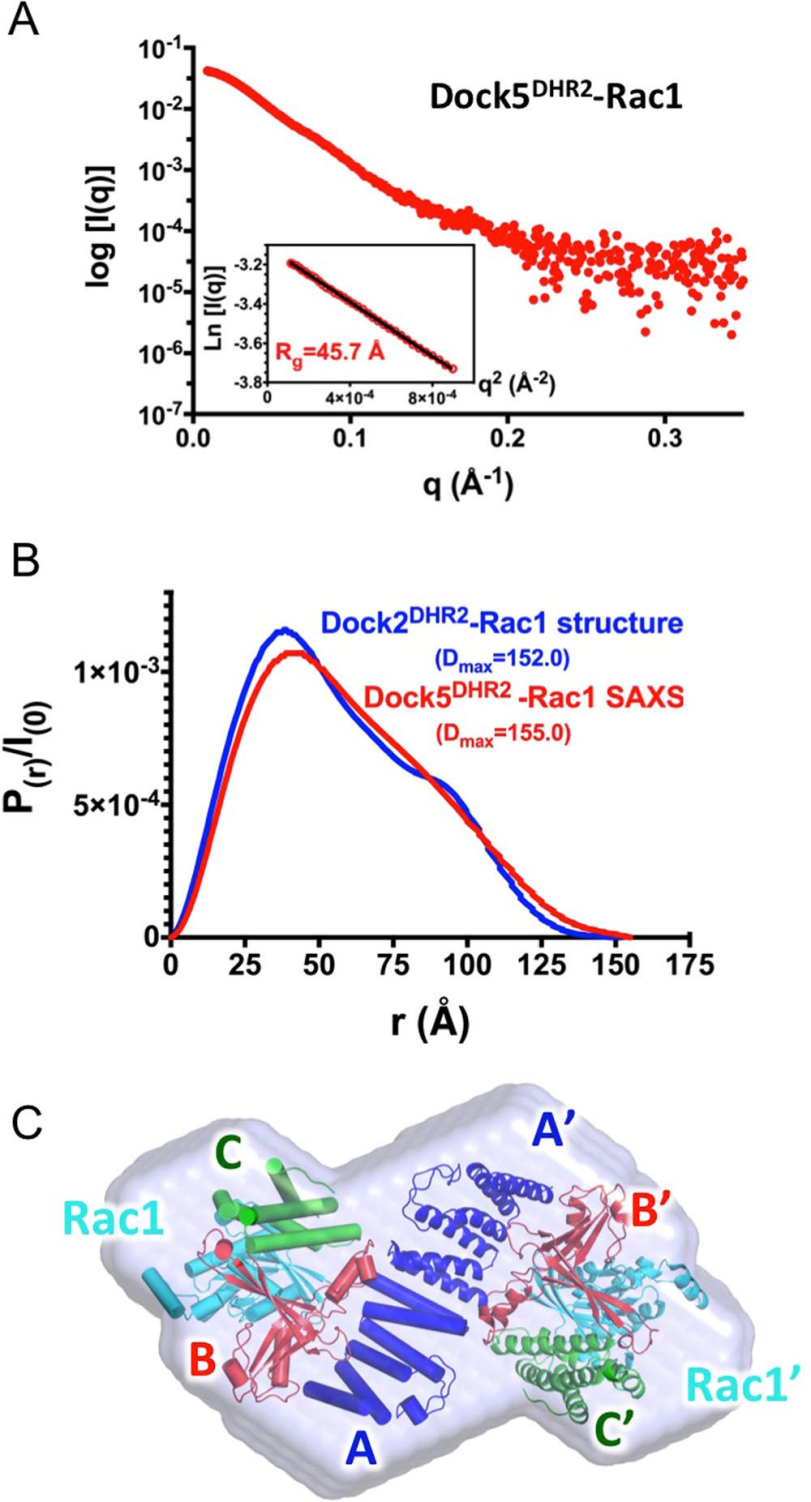
SEC-SAXS analysis of the nucleotide-free Rac1-DOCK5^DHR2^ complex. (**A**) SAXS profile of the DOCK5^DHR2^-Rac1 complex. The insert shows the Guinier plot (q_max_*R_g_ = 1.29) and the estimation of R_g_ from Guinier analysis. (**B**) Comparison of the distance distribution functioI_(r)_/I_(0)_ plots of DOCK5^DHR2^-Rac1 experimental SAXS data (in red) and of Dock2^DHR2^-Rac1 crystal structure (in blue; PDB: 3B13). The D_max_ estimated from the DOCK5^DHR2^-Rac1 SAXS data and calculated from the Dock2^DHR2^-Rac1 crystal structure are indicated. (**C**) Surface representation of the DOCK5^DHR2^-Rac1 model calculated with DAMMIF. The χ^2^ value of the DAMMIF model to the experimental amplitudes is 1.6; the χ^2^ value of the Dock2^DHR2^-Rac1 crystal structure to the DAMMIF model is 3.5. The crystal structure of the Dock2^DHR2^-Rac1 complex was superposed onto the DOCK5^DHR2^-Rac1 envelope using SUPCOMB. Dock2^DHR2^ and Rac1 are represented as in Fig. 1A.

Next, we carried out the SEC-SAXS analysis of nucleotide-free DOCK5^DHR2^-Rac1 (Fig. 4A). For this analysis we used a Rac1 construct lacking the flexible C-terminal peptide and we carefully cleaved the tags used for purification, as these could contribute to the scattering signal in a manner that is difficult to interpret. The R_g_ and D_max_ values are 46 Å and 155 Å, which are almost identical to those calculated from the crystallographic Dock2^DHR2^-Rac1 complex (44 Å and 152 Å) (Fig. 4B). Unlike unbound DOCK5^DHR2^, the R_g_ value of the DOCK5^DHR2^-Rac1 complex was constant across the peak (Figure S3A). The Kratky plot analysis depicts a well-folded structure for DOCK5^DHR2^-Rac1 (Figure S3B). The fit of the experimental data to the amplitudes calculated from Dock2^DHR2^-Rac1 gave a relatively low χ^2^ value of 3.5, indicating that the shapes of the DOCK5^DHR2^-Rac1 and Dock2^DHR2^-Rac1 complexes are similar. These observations suggest that, unlike unbound DOCK5^DHR2^, the DOCK5^DHR2^-Rac1 complex is well represented by a single major conformation. Compared to unbound DOCK5^DHR2^, the DOCK5^DHR2^/Rac1 envelope obtained with DAMMIF displays symmetrical bulges and these extra volumes are readily aligned with the bound GTPase by fitting the crystallographic Dock2^DHR2^-Rac1 into this envelope (Figs 4C and S3C). Altogether, the SAXS analysis of DOCK5^DHR2^ suggests that unbound DOCK5^DHR2^ has conformational dynamics in solution that is restrained by binding of the Rac1 GTPase.

**Figure 5:**
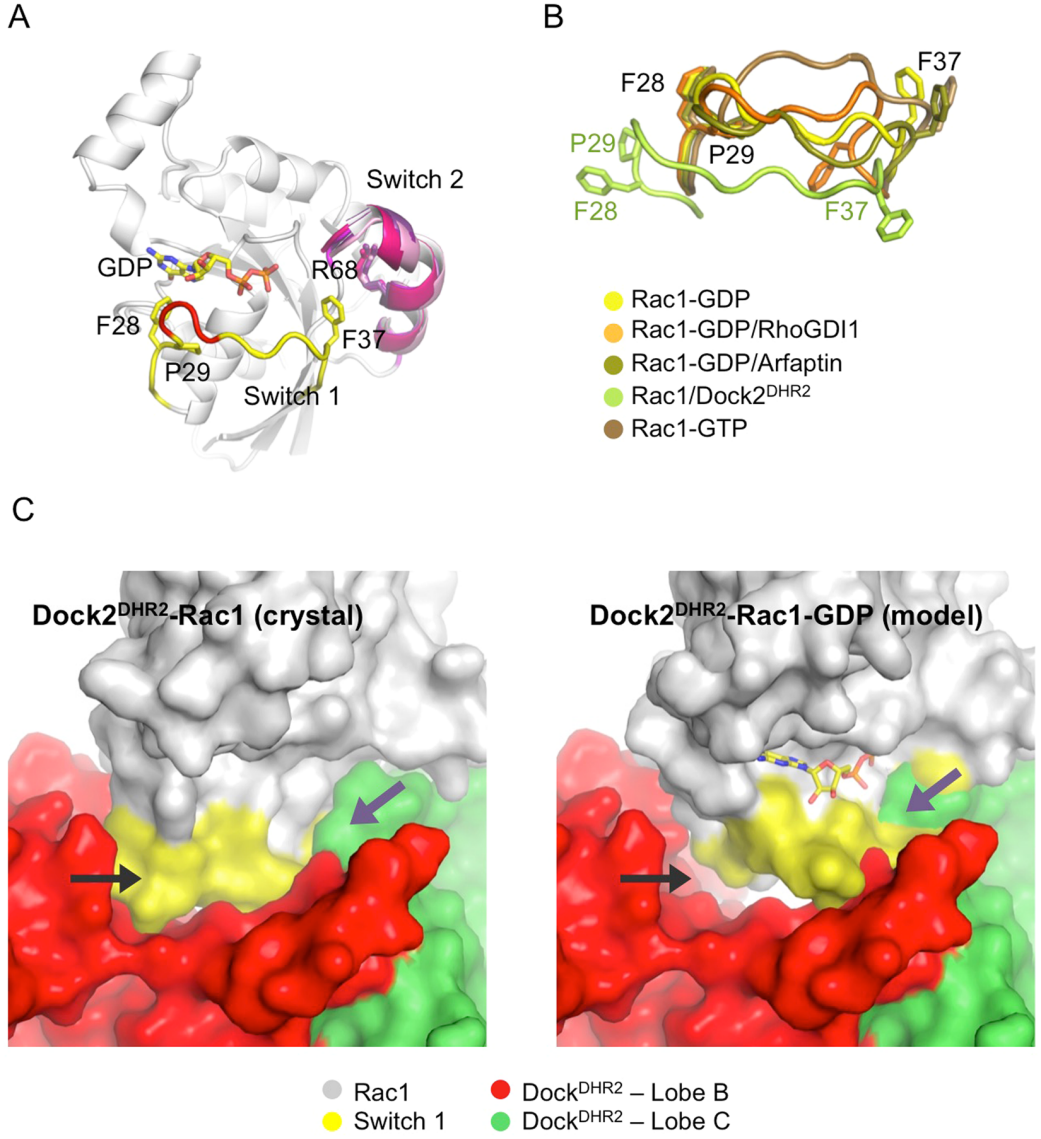
Crystallographic analysis of Rac1-GDP. (**A**) Crystal structure of Rac1-GDP. The switch 2 region is well ordered in Rac1-GDP and its conformation is identical to the switch 2 in Rac1-GDP-RhoGDI1 (PDB entry: 1HH4), Rac1-GDP-Arfaptin (PDB entry: 1I4D), Rac1-Dock2^DHR2^ (PDB entry: 2YIN) and Rac1-GPPNP (PDB entry: 1MH1), overlaid in purple shades. The invariant position of Arg68 is shown for reference. The switch 1 region is in yellow, with poorly ordered residues ^30^GEY^32^ shown in red. (**B**) Close-up view of the overlay of the switch 1 regions in Rac1-GDP, Rac1-GDP-RhoGDI1, Rac1-GDP-Arfaptin, Rac1-Dock2^DHR2^ and Rac1-GPPNP. (**C**) Model of the precatalytic Rac1-GDP-Dock2^DHR2^-bound complex1. Left panel: Nucleotide-free Rac1-Dock2^DHR2^ crystal structure. In this structure, the switch 1 of Rac1 is displaced from the nucleotide-binding site and forms extensive interactions with the B lobe of the DHR2 domain (black arrow). The nucleotide sensor loop is indicated by a violet arrow. Right panel: Rac1-GDP was overlaid on the Rac1-Dock2^DHR2^ complex. The conformation of the switch 1 removes its interactions with α6-β3 in B lobe (black arrow) and generates a steric conflict of ^35^TVF^37^ with the nucleotide sensor loop (violet arrow).

To get further insight into the allosteric inhibition of DOCK5 by C21, we attempted to characterize the solution structure of the DOCK5^DHR2^-C21 complex by SEC-SAXS analysis. However, using a small volume analytic SEC column, we observed a small amount of a larger molecular weight species, which was not detected from the UV elution profiles but appeared in the I_(0)_ analysis and could not be resolved by a computational method. This peak could be resolved by using a small-scale preparative SEC column, although at a large expense on the intensity of the SAXS signal that prevented further analysis (not shown). Although the data measured using both columns have a poorer fit to the DOCK5 model compared to unbound DOCK5, which could suggest that C21 affects the structure of DOCK5^DHR2^, further analysis was not carried out due to these technical limitations. Alternatively, we reasoned that the non-competitive mode of inhibition of C21 indicates that it targets a pre-catalytic Rac1-GDP/Dock^DHR2^ complex that is not yet competent for nucleotide dissociation and must therefore differ from the known crystal structures of small GTPase/Dock^DHR2^ complexes^4,5,7^. To get insight into whether conformational differences between the pre-catalytic and catalytic complexes could be located at the interfaces between Rac1 and the DHR2 domain, we solved the crystal structure of unbound human Rac1-GDP at 2.6 Å resolution (Table 2 **)** and used it to model this Rac1-GDP-Dock2^DHR2^ pre-catalytic intermediate. The conformation of the switch 2 region in unbound Rac1-GDP is well ordered and is the same as in unbound Rac1-GTP^28^ or in the Rac1-Dock2^DHR2^ complex^5^ (Fig. 5A). Thus, while a region important for establishing functional interactions, the switch 2 is formally not a switch region in Rac1 and it can readily engage the C lobe of the DHR2 domain. In contrast, residues ^30^GEY^32^ in the switch 1 of unbound Rac1-GDP are poorly ordered, which drives a unique conformation of the switch 1 that has not been seen in other Rac1 structures (Fig. 5B). When superposed to the Rac1/Dock2^DHR2^ complex, the switch 1 of Rac1-GDP lacks contacts with α6-β3 in the B lobe while residues ^35^TVF^37^ in the switch 1 conflict with the catalytic nucleotide sensor in the C lobe (Fig. 5C). Both the α6-β3 region in the B lobe and the nucleotide sensor in the C lobe are highly conserved between DOCK5 and Dock2^5^. These data suggest that flexibility of the switch 1 supports the formation of a pre-catalytic Rac1-GDP/DOCK5^DHR2^ complex that differs from the catalytically competent intermediate in that the switch1/B lobe interface is not established.

**Table 2:** Crystallographic statistics of unbound Rac1-GDP.

Data collection	
Space group	P2_1_2_1_2_1_
Molecule/a.u.	2
*a*, *b*, *c* (Å)	41.64 82.66 105.90
α, β, γ (°)	90.00 90.00 90.00
Resolution (Å)	44.56–2,59 (2.70–2.59)
*R*_merge_	0.238 (1.279)
*I*/σ	8.4 (1.8)
Completeness (%)	99.5 (99.2)
Redundancy	6.0 (6.0)
**Refinement**	
Resolution (Å)	2.59
Nb. reflections	11862
*R*_work_/*R*_free_	0.193/0,2520
Nb. Atoms (protein)	2885
B-factors (protein) (Å^2^)	32.1
**R**.**m**.**s**. **deviations**	
Bond lengths (Å)	0.004
Bond angles (°)	0.476

We discuss below how C21 may take advantage of these dynamics features in DOCK5 and Rac1-GDP for allosteric inhibition of the reaction.

## Discussion

In this study, we combined biochemical and structural approaches to characterize the biochemical activity of human DOCK5^DHR2^ and its inhibition by small molecules.

Using purified proteins and fluorometric kinetics assays, we found that the DHR2 domain of human DOCK5 is highly active towards Rac1 but does not activate related small GTPases of the Rho/Rac subfamily, and that it is not directly regulated by membranes or by a feed-back mechanism. We also used this approach to characterize two compounds of unrelated chemical structure, C21 and CPYPP, that have been reported to inhibit Dock family members. We found that both compounds inhibit the activation of Rac1 by DOCK5^DHR2^, but only C21 is specific for DOCK5^DHR2^ while CPYPP also inhibits activation of Rac1 by a DH-containing RacGEF. These results establish reference values for the activity, regulation and inhibition of the catalytic DHR2 domain of DOCK5, which should be valuable for future analysis of the regulation of DOCK5 by its other domains and of its manipulation by chemical compounds. Our study emphasizes the usefulness of using pure components and quantitative biochemical assays as a first step towards establishing nucleotide exchange efficiencies, regulations and inhibition of guanine nucleotide exchange factors.

**Figure 6:**
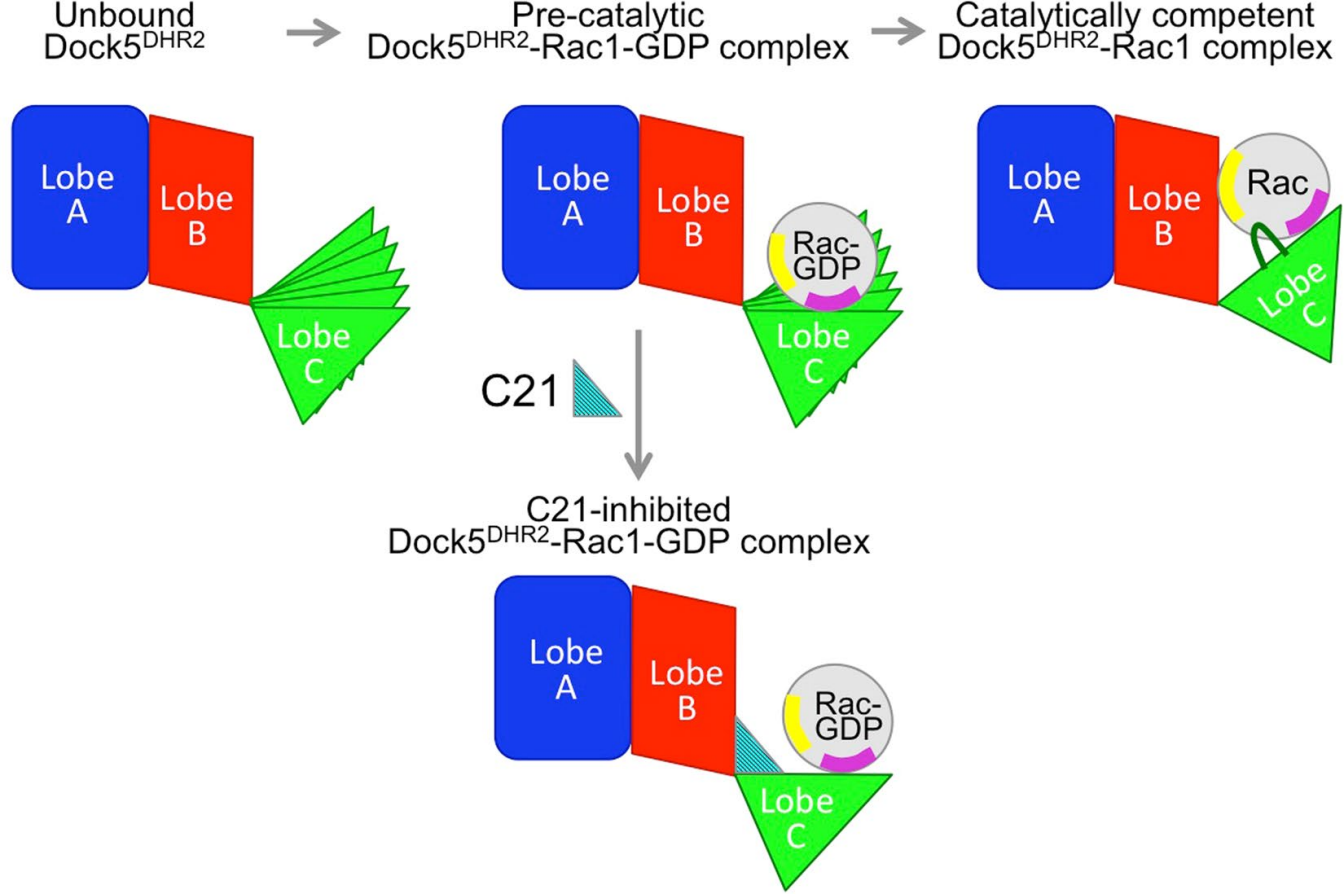
Model of the mechanism of activation of Rac1 by DOCK5^DHR2^ and its inhibition by dynamics diversion. In unbound DOCK5^DHR2^ (top left panel) and in the pre-catalytic DOCK5^DHR2^-Rac1-GDP complex (top, middle panel), the C lobe has intrinsic dynamics. Interaction of Rac1 with the B lobe reduces this flexibility and yields nucleotide dissociation (top, right panel). C21 intercepts a conformation of the C lobe that cannot proceed to the productive complex (bottom panel). See discussion for the details. The A, B and C lobes of the DHR2 domain are indicated. The switch 1 and 2 of Rac1 are indicated in yellow and purple, respectively. The nucleotide sensor loop of the C lobe is shown in dark green.

Our analysis of C21 showed that it inhibits DOCK5-stimulated nucleotide exchange by a non-competitive mechanism, which prompted us to investigate structural features in DOCK5 and Rac1 that could be susceptible to allosteric modulation. We identified two such features: an unexpected conformational dynamics in DOCK5^DHR2^ that diminishes upon Rac1 binding, and a unique conformation of the switch 1 in unbound Rac1-GDP not seen in any of its other conformational states. These data suggest that C21 intercepts a site that forms transiently as the Rac1-GDP/DOCK5^DHR2^ complex visits its conformational landscape. Although our data are not sufficient to map the binding site of C21 or identify structural rearrangements of the C21-DOCK5^DHR2^ complex, they can be aggregated in a simple model (depicted in Fig. 6). In this model, the C lobe of DOCK5^DHR2^ features conformational dynamics that allows Rac1-GDP to bind in successive steps: first, Rac-GDP docks onto the C lobe by its structurally invariant switch 2 region; then, the dynamics of the C lobe brings the switch 1 in contact with the B lobe; finally, this interaction remodels the switch 1 to stabilize the complex, making way for the nucleotide sensor to reach into the nucleotide-binding site and promote the dissociation of GDP. This model suggests an allosteric mechanism in which C21 takes advantage of these dynamical features to stall DOCK5 in an abortive conformation where it can still bind the switch 2 of Rac1-GDP but cannot escort it to the B lobe. Future experiments will be needed to determine the precise nature of intramolecular dynamics in the exchange reaction and how they are modulated by inhibitors.

Inhibitors of protein-protein interactions have tremendous therapeutic potential but they have remained challenging to discover, notably because small chemical compounds are ill-suited to interrupt large protein-protein interfaces, whether by competitive or allosteric mechanisms (reviewed in ref.^29^). Allosteric inhibitors that do not block protein-protein interactions but instead remodel protein-protein complexes into unproductive conformations represent a promising alternative to chemical disrupters. An illuminating example is that of Brefeldin A (BFA), a natural compound that inhibits the activation of small GTPases of the Arf family. BFA binds at the interface between the GTPase and its GEF in the course of the exchange reaction, thus stalling the GTPase-GEF complex in a conformation that cannot proceed to GDP dissociation^30^. BFA is the founding representative of interfacial inhibitors, which take advantage of functional remodeling of protein complexes to stabilize abortive conformations resulting in inhibition of cellular and *in vivo* functions^31^. However, a challenge in understanding conformational dynamics and exploiting them for drug discovery is that their analysis by structural methods and docking simulations has remained arduous^32^. Our study shows that SAXS can complement more classical approaches, such as NMR, to gain insight into structural dynamics relevant to the inhibition of protein interactions. The mode of action of C21 identified here expands the repertoire of allosteric inhibitory mechanisms whereby protein-protein interactions are inhibited without disruption thus effectively blocking processes controlled by the complex in cells and *in vivo*. We propose that diversion of intramolecular dynamics is a general Achille’s heel that can be exploited by chemical compounds to remodel protein complexes into unproductive conformations, with potential for a variety of applications to the inhibition of regulatory and hub proteins in diseases.

## Material and Method

### Cloning, protein production and purification

Expression and purification of human full-length Rac1, Rac2, RhoA, RhoG and CDC42 carrying a C-terminal 6-histidine tag and of human Trio^DH1−PH1^ (residues R1232-T1550) are as in ref.^22^. The DHR2 domain of human DOCK5 (residues I1212-L1642) carrying a 6xHis-MBP tag followed by a TEV protease cleavage site was cloned into the Gateway destination vector pHMGW. Human Rac1 lacking residues 177–192 and carrying a 6xHis tag in N-terminus followed by a TEV protease cleavage site was cloned into pET28a. All clones were confirmed by sequencing (GATC Biotech).

All proteins were produced in Rosetta (DE3) pLysS *Escherichia coli* strains in LB medium by induction with 0.5 mM IPTG overnight at 20 °C. Bacterial pellets were resuspended in lysis buffer (20 mM Tris pH 8.0, 500 mM NaCl, 2 mM β-mercaptoethanol, 2 mM MgCl_2_, 10% glycerol, 0.5% tween-20, anti-protease cocktail) and frozen in liquid nitrogen. After thawing, benzonase was added to 7.5 U/mL and cells were disrupted using a French press, cleared by centrifugation at 14 000 g for 30 minutes and the supernatant was filtered over a 0.22 μm filter. Proteins were first purified by an affinity step using a 5 mL MBP-Trap column (GE Healthcare) with elution at 10 mM maltose for DOCK5^DHR2^ or a His-trap column (GE Healthcare) with elution at 500 mM imidazole for Rac1, followed by gel filtration on a Superdex 200 column (GE Healthcare) equilibrated with 20 mM Tris pH 8.0, 500 mM NaCl, 2 mM β-mercaptoethanol, 2 mM MgCl_2_, 10% glycerol. All proteins eluted as a single and symmetrical peak. Fusion tags were cleaved with the His-TEV protease (1:20 w/w) overnight at 4 °C, then samples were reloaded on a Talon Crude column (GE Healthcare). The purity of the non-retained fraction containing the cleaved protein was polished by gel filtration on a Superdex 200 column equilibrated with 20 mM Tris pH 8.0, 500 mM NaCl, 2 mM β-mercaptoethanol, 10% glycerol, 2 mM MgCl_2_. Protein purity was assessed by SDS-PAGE (Fig. 1B).

### GEF assay using fluorescence kinetics

Nucleotide exchange kinetics was measured by recording the decay in fluorescence following the dissociation of Mant-GDP pre-loaded onto the small GTPase (λ_Ex_ = 360 nm, λ_Em_ = 440 nm) as described^16,22^. The concentration of small GTPases was 1.0 μM in all experiments. The concentration of DOCK5^DHR2^ or Trio^DH1-PH1^ was 100 nM for single k_obs_ (s^−1^) determinations. For k_cat_/K_M_ (M^−1^s^−1^) determinations, the concentrations of DOCK5^DHR2^ ranged from 25 nM to 250 nM. For exchange assays carried out in the presence of liposomes, the concentration of total lipids was 100 μM. For the feedback experiment, DOCK5^DHR2^ was incubated with 1.0 μM of GTPγS-loaded GTPases prior to measuring nucleotide exchange. K_obs_ and k_cat_/K_M_ values were determined by single exponential analysis as described in ref.^21^. For the inhibition studies, inhibitors were used at 25 μM. Analysis of kinetic traces by a single exponential was preferred over initial velocities, which can be affected by chemical compounds intrinsic fluorescence. Error bar represent standard deviations. All experiments were done at least in triplicate.

### Liposome flotation assays

Sucrose-loaded liposomes containing 46% PC, 20% PE, 10% PS, 2% PI(4,5)P_2_, 20% cholesterol, 2% DOG-NiNTA lipids were prepared as described^33^. Full-length GTPases (2.0 μM) were incubated with 100 μM GDP and were tethered to Ni-lipid containing liposomes (1 mM total lipids) by their 6xHis tag. The concentration of DOCK5^DHR2^ was 2.0 μM in all experiments. The liposome flotation experiments were performed as described in refs^33,34^. Liposome-bound proteins (top fraction) and unbound proteins (bottom fraction) were collected and analyzed by SDS-PAGE. All experiments were done in triplicate.

### Gel filtration experiments

All experiments were carried out in elution buffer (20 mM Tris pH 8.0, 150 mM NaCl, 0.5 mM EDTA) containing either 25 μM C21 diluted from a DMSO stock or the equivalent amount of DMSO. This is the maximum concentration at which the inhibitor is soluble. DOCK5^DHR2^ was used at 25 μM in all experiments. DOCK5^DHR2^ was pre-incubated with DMSO or 25 μM C21 for 30 min, before addition of truncated Rac1 (50 μM) and 10 mM EDTA to favor the formation of the nucleotide-free complex. Proteins samples were injected onto a Superdex 200 gel filtration column (GE-Healthcare). The concentration of DOCK5^DHR2^ at the elution peak is estimated to ≈ 1 μM, ensuring that the inhibitor is in ≈ 25-fold excess.

### Chromatography-coupled SAXS data acquisition

All SAXS data were obtained from synchrotron SEC-SAXS experiments. DOCK5^DHR2^ was used at a concentration of 80 μM to obtain high SAXS intensities. Samples were injected on a high-performance liquid chromatography (HPLC) size exclusion column (BioSEC-3 300 Å, Agilent Technologies, Inc.) equilibrated with the elution buffer (20 mM Tris pH 8.0, 150 mM NaCl, 0.5 mM EDTA). For the nucleotide-free Rac1/DOCK5^DHR2^ complex, DOCK5^DHR2^ was pre-incubated with a 2-fold excess of Rac1^1–177^ and 10 mM EDTA. For SEC-SAXS experiments of the C21/DOCK5^DHR2^ complex, DOCK5^DHR2^ was pre-incubated with 25 μM C21, and the elution buffer was supplemented with 25 μM C21. For this set of experiments, we used either a small volume analytical SEC column (BioSEC-3) or a small-scale preparative SEC column (Superdex-200 Increase, GE Healthcare). SAXS experiments were conducted on the SWING beamline at the SOLEIL synchrotron essentially as described in ref.^35^. Data reduction to absolute units, frame averaging, and subtraction were done using the FOXTROT program (synchrotron SOLEIL). Frames corresponding to the high-intensity fractions of the peak and having constant *R*_g_ within error were averaged.

### SAXS Data Analysis and Structural Modeling

All the data analyses were performed with programs from the ATSAS package^36^ unless specified otherwise and are summarized in Table 1. Radii of gyration (R_g_) were evaluated by Guinier Wizard using the data within the range of Guinier approximation sR_g_ < 1.3 and by Distance Distribution Wizard, both of which are modules of the PRIMUS program. The maximum distance D_max_ was estimated with PRIMUS and refined by trial and error with GNOM. The distance distribution functions P_I_ were calculated with GNOM. For DOCK5^DHR2^, we determined a range of D_max_ values that accounted well for the experimental SAXS data. We then calculated DAMMIF models (see below) using D_max_ values within this range, and selected the smallest D_max_ value that accounted for the known 2-fold symmetry of DOCK5^DHR2^. The dimensionless Kratky plot was calculated by plotting (qR_g_)^2^I_(q)_/I_(0)_ against qR_g_^37^. The molecular weights of the proteins were estimated by the ScÅtter program (q = 0.25)^38^. The fit between scattering amplitudes calculated for crystal structures and the SAXS profiles were carried out with CRYSOl. *ab initio* shape determinations and structural envelopes calculations were done by DAMMIF, using the SAXS data within a *q* range of 0.01–0.2 Å^−1^. Initial calculations were carried out without imposing P2 symmetry and this resulted in models that were visually symmetrical. Given that all Dock^DHR2^ proteins with known crystal structures are symmetrical dimers and DOCK5^DHR2^ is itself a dimer, subsequent models were constrained by a P2 symmetry to improve the quality of the models. 20 independent runs were carried out for each dataset. The resulting models were further compared and clustered by SUPCOMB. The Normalized Spatial Discrepancy (NSD) values were close to 1, which is in the high end for acceptable similarity between models but likely reflects the anisotropy of the structure. The model with the best fit to the experimental data was chosen for comparison with the crystal structure of Dock2^DHR2^. The PDB coordinates of the crystal structures were superimposed onto the SAXS-constructed envelopes of the proteins by SUPCOMB. SAXS data sets will be deposited with the small angle scattering biological data bank SASBDB (https://www.sasbdb.org/).

### Crystallographic analysis

Crystallization screens were performed using the sitting-drop vapor diffusion method at 18 °C with a Mosquito robot (TTP LabTech) in 96-well crystallization plates by mixing 100 nL of Rac1^1–177^-GDP at 5 mg/mL solution with 100 nL of precipitant solution. Crystals were obtained in 0.1 M MES pH 6.5 and 20% PEG 3350. Crystals were cryo-protected using the reservoir solution supplemented with 10% glycerol prior to flash freezing. A complete diffraction dataset was collected on PROXIMA-2A beamline (SOLEIL synchrotron, France) and was integrated with the program XDSme (https://code.google.com/archive/p/xdsme/). The structure was solved by molecular replacement with Phaser^39^ using Rac1-GPPNHP^28^ as a model. Refinement was carried out with the program BUSTER^40^ using TLS parameters, in alternation with graphical building using Coot^41^. The quality of the structure was assessed using MolProbity^42^. Data collection and refinement statistics are reported in Table 2. Coordinates and structure factors have been deposited in the Protein Data Bank with entry code 5N6O.

## Electronic supplementary material

Supplementary captions and figures
