# Supplementary material for: Allosteric inhibition of the guanine nucleotide exchange factor DOCK5 by a small molecule
Source: Sci Rep. 2017 Oct 31;7:14409. doi: 10.1038/s41598-017-13619-2 (PMC5663973; doi:10.1038/s41598-017-13619-2)
Supplement: Supplementary file 1 — Supplementary captions and figures [file 41598_2017_13619_MOESM1_ESM.pdf]

Allosteric inhibition of the guanine nucleotide exchange factor Dock5 by a small molecule

Yann Ferrandez, Wenhua Zhang, François Peurois, Lurlène Akendengué, Anne Blangy, Mahel Zeghouf, Jacqueline Cherfils

SUPPLEMENTARY FIGURE LEGENDS

Figure S1. Dock5^{DHR2} forms a stable complex with Rac1-GDP and Rac1-GTP

Size exclusion chromatography of Rac1 alone, Dock5^{DHR2} alone, Dock5^{DHR2} with Rac1-GTP and Dock5^{DHR2} with Rac1-GDP, as indicated. Experiments were carried out in elution buffer (20 mM Tris pH 8.0, 150 mM NaCl, 1mM $MgCl_2$). Dock5^{DHR2} was used at 80 μM. Rac1 (truncated form, 160 μM) was preloaded with GTP or GDP. Proteins were injected onto a Superdex 200 gel filtration column (GE-Healthcare). Right panel: The peaks containing the complexes (indicated by an arrow) were analyzed by SDS-PAGE.

Figure S2. SEC-SAXS data acquisition, folding analysis and DAMMIF model of Dock5^{DHR2}.

A. SEC-SAXS analysis. Frame-resolved $I_{(0)}$ and R_g estimation in the course of the HPLC-SEC elution. Frames 160-172 were selected for data averaging.

B. Dimensionless Kratky plot.

C. Structural alignment of the Dock2^{DHR2} dimer crystal structure (shown in cartoon) onto the Dock5^{DHR2} SAXS model (shown as envelope). The view is rotated by 90 degrees with respect to **Figure 2D**.

Figure S3. SEC-SAXS data acquisition, folding analysis and DAMMIF model of the Dock2^{DHR2}-Rac1 complex.

A. SEC-SAXS analysis. Frame-resolved $I_{(0)}$ and R_g estimation in the course of the HPLC elution. Frames 185-205 were selected for data averaging.

B. Dimensionless Kratky plot.

C. Structural alignment of the Dock2^{DHR2}-Rac1 complex crystal structure (shown in cartoon) onto the SAXS model of Dock5^{DHR2}-Rac1 complex (shown as envelope). The view is rotated by 90 degrees with respect to **Figure 3D**.

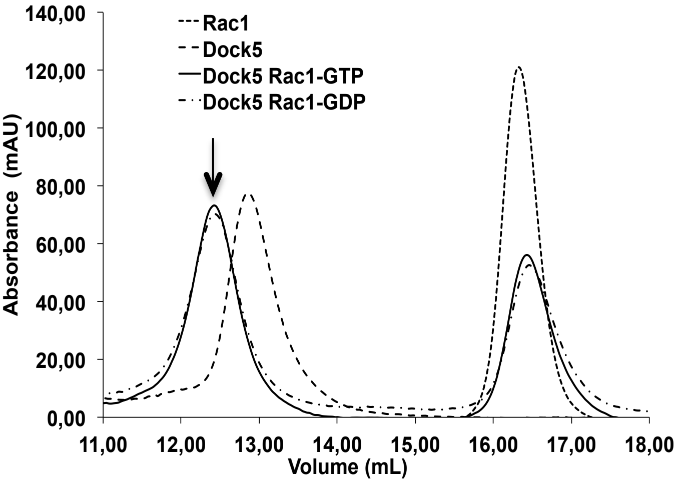

140,00
120,00
100,00
80,00
60,00
40,00
20,00
0,00
Absorbance (mAU)
Rac1
Dock5
Dock5 Rac1-GTP
Dock5 Rac1-GDP
11,00
12,00
13,00
14,00
15,00
16,00
17,00
18,00
Volume (mL)

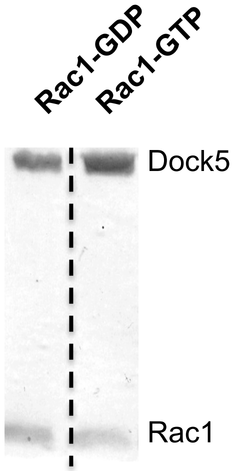

Rac1-GDP
Rac1-GTP
Dock5
Rac1

Figure S1

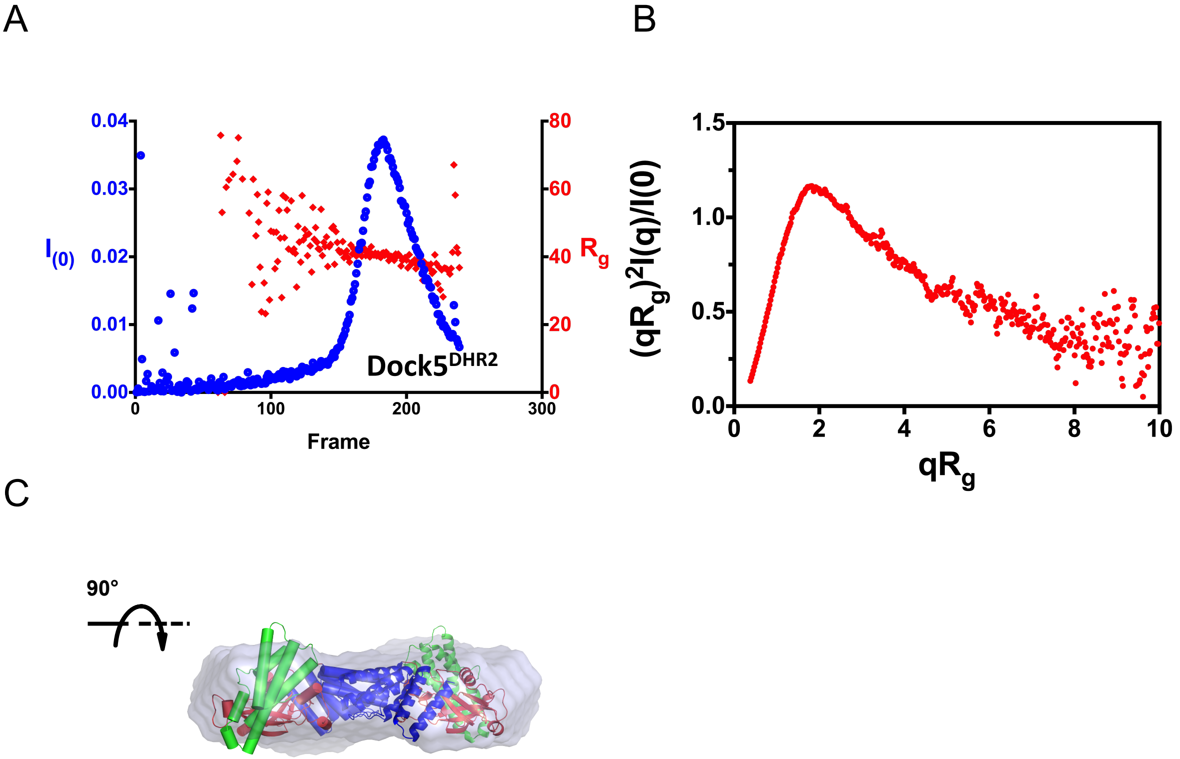
A
B
C
0.04
0.03
0.02
0.01
0.00
$I_{(0)}$
80
60
40
20
0
R_g
0
100
200
300
Frame
Dock5[DHR2]
1.5
1.0
0.5
0.0
$(qR_g)^2I(q)/I(0)$
0
2
4
6
8
10
qR_g
90°

Figure S2

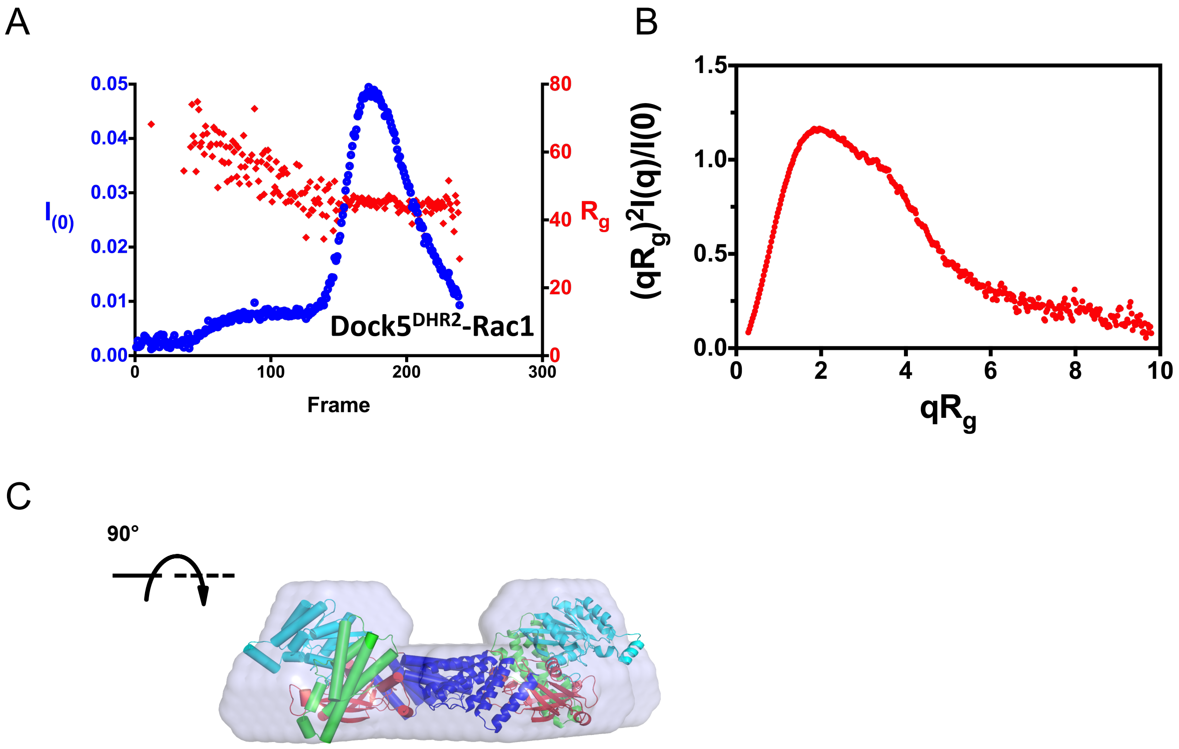
A
0.05
0.04
0.03
0.02
0.01
0.00
I(0)
80
60
40
20
0
Rg
0
100
200
300
Frame
Dock5DHR2-Rac1
B
1.5
1.0
0.5
0.0
(qRg)2I(q)/I(0)
0
2
4
6
8
10
qRg
C
90°

Figure S3
